# A thermostable bacterial lytic polysaccharide monooxygenase with high operational stability in a wide temperature range

**DOI:** 10.1186/s13068-020-01834-5

**Published:** 2020-11-30

**Authors:** Tina Rise Tuveng, Marianne Slang Jensen, Lasse Fredriksen, Gustav Vaaje-Kolstad, Vincent G. H. Eijsink, Zarah Forsberg

**Affiliations:** Faculty of Chemistry, Biotechnology and Food Science, NMBU-Norwegian University of Life Sciences, Aas, Norway

**Keywords:** LPMO, Thermostability, Cellulose, Synergy

## Abstract

**Background:**

Lytic polysaccharide monooxygenases (LPMOs) are oxidative, copper-dependent enzymes that function as powerful tools in the turnover of various biomasses, including lignocellulosic plant biomass. While LPMOs are considered to be of great importance for biorefineries, little is known about industrial relevant properties such as the ability to operate at high temperatures. Here, we describe a thermostable, cellulose-active LPMO from a high-temperature compost metagenome (called mgLPMO10).

**Results:**

MgLPMO10 was found to have the highest apparent melting temperature (83 °C) reported for an LPMO to date, and is catalytically active up to temperatures of at least 80 °C. Generally, mgLPMO10 showed good activity and operational stability over a wide temperature range. The LPMO boosted cellulose saccharification by recombinantly produced GH48 and GH6 cellobiohydrolases derived from the same metagenome, albeit to a minor extent. Cellulose saccharification studies with a commercial cellulase cocktail (Celluclast®) showed that the performance of this thermostable bacterial LPMO is comparable with that of a frequently utilized fungal LPMO from *Thermoascus aurantiacus* (*Ta*LPMO9A).

**Conclusions:**

The high activity and operational stability of mgLPMO10 are of both fundamental and applied interest. The ability of mgLPMO10 to perform oxidative cleavage of cellulose at 80 °C and the clear synergy with Celluclast® make this enzyme an interesting candidate in the development of thermostable enzyme cocktails for use in lignocellulosic biorefineries.

## Background

Lignocellulosic biomass (i.e., plant-based biomass) is the most abundant source of renewable carbon on Earth [1] and its major component is cellulose, a linear polymer of glucose units joined by β-1,4-glycosidic bonds [2]. Individual cellulose chains commonly arrange into crystalline fibrils consisting of multiple chains that are stabilized by extensive hydrogen bonding, and this assembly makes cellulose highly resistant to both chemical and microbial degradation [3, 4]. However, certain microorganisms have evolved to overcome this recalcitrance by producing specialized enzymes, and these may be employed in industrial bioprocessing for the sake of generating renewable energy and bulk commodities [5, 6].

Cellulose-degrading enzymes include cellulases that cleave glycosidic bonds using a hydrolytic mechanism, which are further classified as endoglucanases or exo-acting cellobiohydrolases (CBHs). While endoglucanases are thought to attack randomly in amorphous (i.e., loosely packed) cellulose by engulfing the cellulose chain in a catalytic cleft, cellobiohydrolases attack the cellulose from accessible chain ends using a tunnel-shaped active site [7]. The turnover of cellulose by cellulases is hampered by the fact that the enzymes cannot easily access the crystalline surfaces of the cellulose fibrils [8].

Enzymes called lytic polysaccharide monooxygenases (LPMOs) employ a powerful oxidative mechanism to cleave glycosidic bonds within crystalline regions of densely packed polysaccharides such as cellulose and chitin [8–10]. This mode of action is enabled by a flat substrate-binding surface with a surface-exposed active site. The ability of LPMOs to disrupt crystalline cellulose fibrils, thereby granting the hydrolytic enzymes access to binding sites in parts of the substrate that they would otherwise struggle to reach [9, 11], makes LPMO activity crucial in the development of industrial bioprocessing technologies [12, 13].

The catalytic function of LPMOs was discovered in 2010 [9] and today these enzymes are classified as auxiliary activities (AA) in the carbohydrate-active enzymes database (http://www.cazy.org [14]). Currently, LPMOs are categorized in CAZy families AA9-11 and AA13-16 on the basis of sequence similarity, and may be active on cellulose, various types of hemicelluloses, chitin, starch and/or oligosaccharides [10, 15]. Thus, LPMOs hold an important role in the Earth’s carbon cycle.

LPMOs hydroxylate either the C1 or C4 carbon of the scissile glycosidic bond in cellulose [9, 16, 17], whereas some are less regioselective and produce a mixture of C1- and C4-oxidized products. Oxidation of the C1 carbon results in the formation of 1,5-δ-lactones that are spontaneously hydrated to the more stable aldonic acid form, while oxidation of the C4 carbon produces 4-ketoaldoses that are hydrated to their corresponding gemdiol form [18]. Like cellulases, LPMOs may be associated with one or more carbohydrate-binding modules (CBMs), often connected via a flexible linker [19].

Two highly conserved histidines located in the center of the flat substrate-binding surface constitute the catalytic core of LPMOs. The histidines coordinate a single copper ion, which must be reduced from the Cu(II) to the Cu(I) state before the LPMO can initiate oxidative cleavage [17]. Notably, reduced LPMOs are known to be prone to oxidative damage when exposed to O_2_ or H_2_O_2_ in the absence of a proper substrate [20–22].

LPMO catalysis has generally been thought to be strictly dependent on molecular oxygen and a reductant that delivers two electrons and two protons for each catalytic cycle [9, 23, 24]. However, recent studies on LPMOs belonging to families AA9 and AA10, have shown that H_2_O_2_ can drive LPMO reactions, and that these reactions are orders of magnitude faster than O_2_-driven reactions [20, 25–29]. The peroxygenase driven reaction only requires sub-stoichiometric amounts of reductant for an initial, “priming”, reduction of the LPMO, after which the enzyme can catalyze multiple reactions. Notably, under the conditions normally used in LPMO reactions, H_2_O_2_ will be formed either by non-productive oxidase activity of reduced LPMO molecules that are not bound to the substrate [18, 30] or by reactions involving dioxygen and the reductant.

Although LPMOs have been intensely studied since their discovery in 2010 [10, 15, 22, 31], and even though they are considered to be of great importance for industrial biorefining [12, 13], little is known about industrially relevant properties such as activity and stability at higher temperatures. Thermostability is a commonly desired trait for enzymes employed in biorefineries because stable enzymes last longer and because it is often considered favorable to run processes at higher temperatures, for example to improve reactant solubility and reduce the risk of microbial contamination [32]. Naturally occurring thermostable enzymes can be discovered by bioprospecting of high-temperature environments where biomass turnover occurs in Nature. To date, only a few studies have aimed at engineering thermal stability of LPMOs [33, 34].

The present study describes the characterization of a thermostable bacterial AA10 LPMO with a C-terminal CBM2 domain. The gene encoding the enzyme has previously been identified as the only LPMO overexpressed in a metatranscriptome originating from rice straw that was inoculated with compost and incubated at high temperature [35]. We have compared the properties of this LPMO, named mgLPMO10 (mg, for metagenome), with the properties of a similar two-domain mesophilic LPMO from *Streptomyces coelicolor*, *Sc*LPMO10C [36], and we have assessed its potential to boost the activity of various cellulases.

## Results

### Verification of activity and thermal stability

**Fig. 1:**
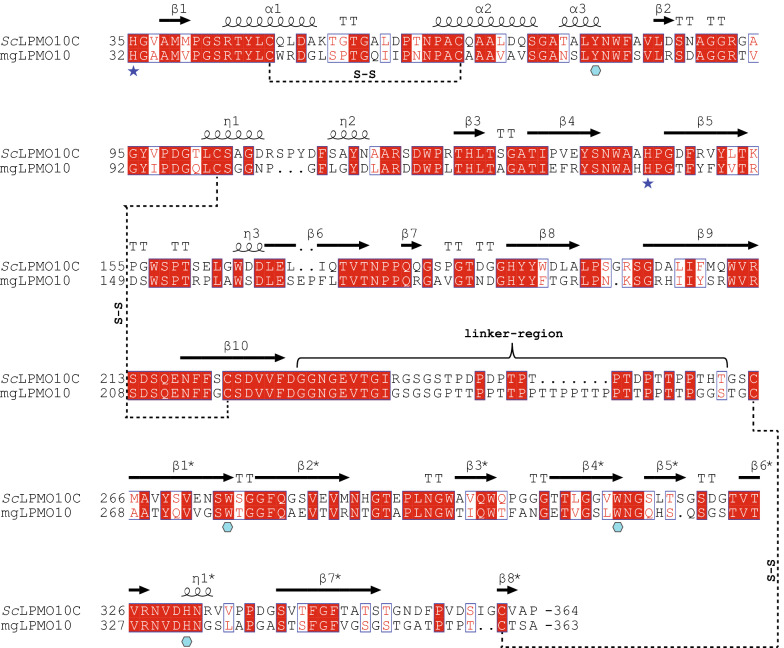
Sequence alignment of full-length *Sc*LPMO10C and mgLPMO10. The secondary structure shown above the alignment was obtained from the crystal structure of the AA10 domain of *Sc*LPMO10C (PDB ID: 4OY7; [36]) and the NMR structure of the CBM2 domain (PDB ID: 6F7E, [19]). The two catalytic histidines (*Sc*LPMO10C His35 and His144 and mgLPMO10 His32 and His138) are labeled with blue stars. Three disulfide bridges present in *Sc*LPMO10C, two in the AA10 module and one in the CBM2, are indicated by dashed lines; note that the six cysteine residues are all conserved in mgLPMO10. Residues that have been shown to be important for cellulose binding by *Sc*LPMO10C [19] (Tyr79, Trp275, Trp312 and His331) are shown with turquoise hexagons. Fully conserved residues are highlighted in red, while blue boxes indicate non-conserved residues with similar properties

**Fig. 2:**
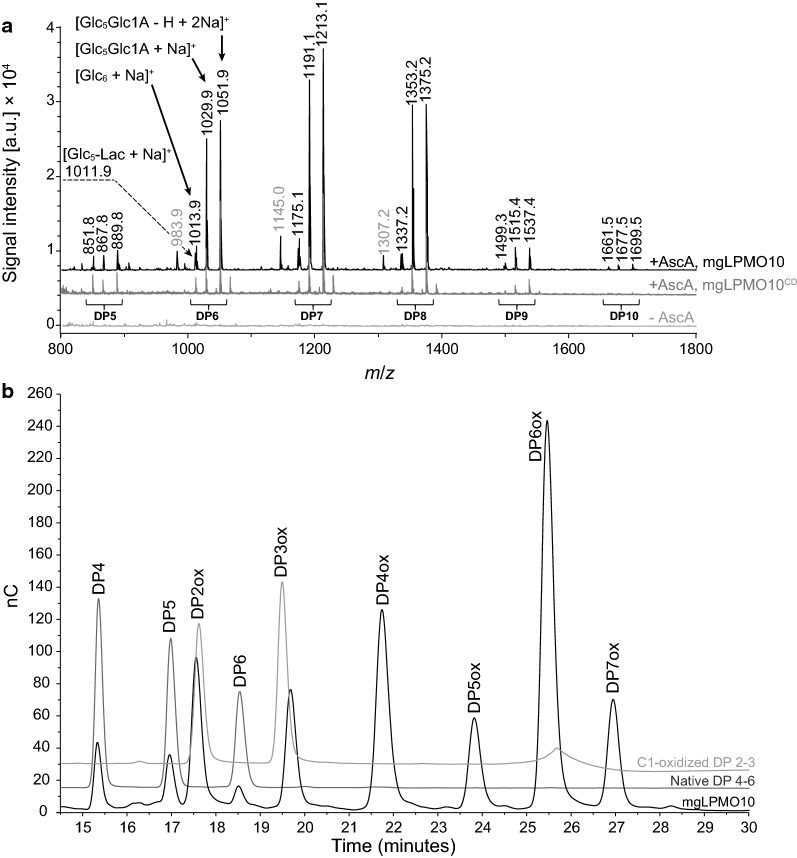
Verification of LPMO activity. Full-length mgLPMO10 or mgLPMO10^CD^ was incubated with 5 g/L PASC or 10 g/L Avicel in 50 mM sodium phosphate buffer (pH 6.0) supplemented with 1 mM ascorbic acid at 60 °C for 24 h. The supernatant was subjected to analysis by MALDI-ToF (PASC samples) for both mgLPMO10 variants (**a**) and HPAEC-PAD (Avicel samples) for full-length mgLPMO10 (**b**). The peaks of the hexamer cluster are denoted by arrows and show the sodium adduct of native cellohexaose [Glc_6_ + Na]^+^ and two larger peaks that represent sodium adducts of C1-oxidized cellohexaose (aldonic acid), namely [Glc_5_Glc1A + Na]^+^ and [Glc_5_Glc1A − H + 2Na]^+^. Note that the 1,5-δ-lactone (*m*/*z* − 2 species) is also visible at 1011.9, labeled [Glc_5_-Lac + Na]^+^. Of note, the spectrum shows a series of minor signals (984, 1145, 1307, grey-labeled masses) differing by one glucose (162 m*/z*) that represent products of unknown nature. No products were observed for a reaction with only PASC and AscA, and neither for a reaction with PASC and the LPMO but in absence of AscA (grey spectrum). The HPAEC chromatogram for mgLPMO10 shows similar product profile as the well-characterized *Sc*LPMO10C with C1-oxidized cello-oligomers ranging from DP 2-7. No C4-oxidized products, that have longer retention times [18], were detected

The full-length LPMO (mgLPMO10; residues 32–363) and the catalytic domain (mgLPMO10^CD^; residues 32–223) were expressed and purified to electrophoretic homogeneity. The yield for mgLPMO10 was typically around 12–15 mg purified protein per liter of *E. coli* and 4–5 mg per liter *E. coli* culture for mgLPMO10^CD^. We also purified *Sc*LPMO10C, a C1-oxidizing cellulose-active LPMO [37], which was included in the experiments to enable comparison of mgLPMO10 to a mesophilic homologue. The overall sequence identity between full-length *Sc*LPMO10C and mgLPMO10 is 56%; 62% between the catalytic AA10 domains and 47% between the CBM2 domains (Fig. 1). The LPMO and CBM2 domains are separated by a proline- and threonine-rich linker that is seven residues longer in mgLPMO10. The closest homologue of mgLPMO10, as identified by a BLAST search, is an uncharacterized putative LPMO from *Micromonospora* sp. HM5-17, which shares 85% and 95% sequence identity, for the full-length protein and the catalytic domain, respectively, suggesting mgLPMO10 to be of Actinomycetales origin. The oxidative activity of mgLPMO10 on cellulose was initially verified using PASC and Avicel under standard conditions (e.g., 1 mM ascorbic acid, aerobic conditions). The MALDI-ToF product profile showed peaks characteristic for oxidized cello-oligosaccharides (black spectrum), including peaks representing the sodium salt of the sodium adduct of the aldonic acids (*m*/*z* + 38 species), which (in combination with very low levels of the *m*/*z* -2 species) is diagnostic for C1-oxidation (Fig. 2a). The regioselectivity was confirmed by HPEAC-PAD analysis of product mixtures, which showed only C1-oxidized products (Fig. 2b).

**Fig. 3:**
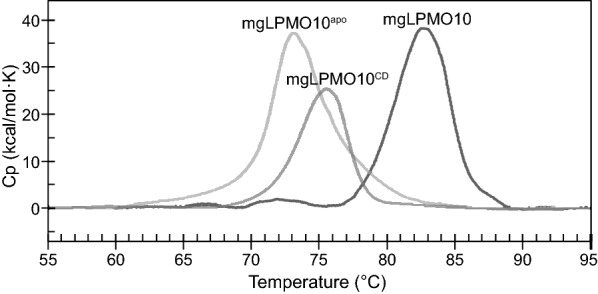
Thermal unfolding of mgLPMO10. The apparent melting temperatures (*T*_m(app)_) were determined by differential scanning calorimetry (DSC) by heating the full-length mgLPMO10-*holo* (approximately 0.4 g/L) at 1 °C/min from 25 °C to 100 °C in 50 mM sodium phosphate buffer (pH 6.0). The experiment was repeated for the *apo* version of the protein (mgLPMO10^apo^) and the *holo* catalytic domain (mgLPMO10^CD^)

Differential scanning calorimetry (DSC; Fig. 3) showed that the *T*_m(app)_ of the full-length mgLPMO10, is approximately 83 °C, and that truncation of the linker and the CBM2 ([*T*_m(app)_] 76 °C) as well as removal of the copper cofactor (*T*_m(app)_ 73 °C) have negative effects on the thermal stability. All unfolding events showed a single transition and unfolding was irreversible in all cases.

### The effect of temperature on LPMO activity

**Fig. 4:**
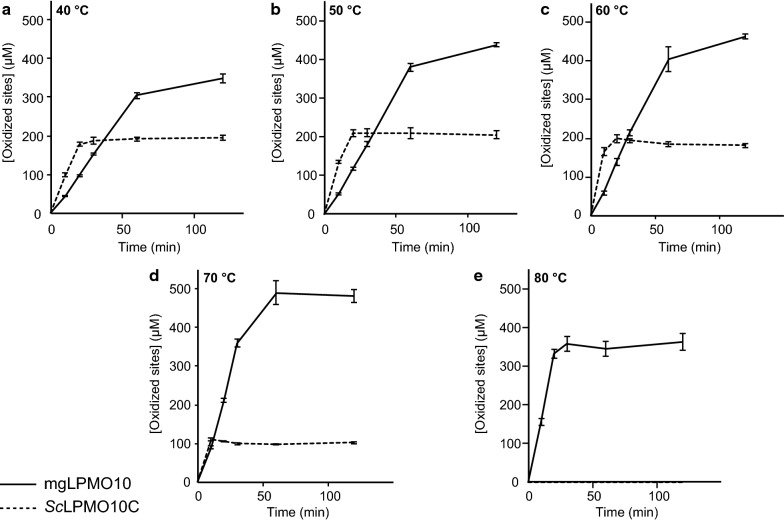
Activity of mgLPMO10 and *Sc*LPMO10C at various temperatures. **a**–**e** show progress curves for copper-saturated mgLPMO10 (solid curves) and *Sc*LPMO10C (dashed curves) at 40, 50, 60, 70 and 80 °C, respectively. The reactions were carried out with 1 µM LPMO and 10 g/L Avicel in 50 mM sodium phosphate buffer pH 6.0. The reactions were pre-incubated for 10 min at various temperatures before 1 mM ascorbic acid was added to start the LPMO reaction. The reactions were carried out in Eppendorf Thermomixers at 800 rpm and samples were taken and filtered at 10, 20, 30, 60 and 120 min. The solubilized products were degraded to oxidized dimers and trimers by incubation with 0.5 µM mgCel6A prior to product quantification. The reaction with *Sc*LPMO10C at 80 °C (**e**) did not yield any oxidized products. The error bars show ± SD (*n* = 3)

Monitoring of product formation over time at varying incubation temperatures (Fig. 4) showed a trade-off between activity and enzyme inactivation and revealed a clear difference between mgLPMO10 and *Sc*LPMO10C. For mgLPMO10, the initial activity increased with temperature all the way up to 80 °C (the highest tested temperature). Only at 80 °C, the product formation curve showed clear signs of enzyme inactivation, with product levels stabilizing already after approximately 20 min at a level (approximately 350 μM solubilized product) that is lower than the highest observed product level (approximately 500 μM in the reaction carried out at 70 °C). In contrast, *Sc*LPMO10C showed clear signs of enzyme inactivation in the reaction carried out at 70 °C (Fig. 4), which is about 6 °C above its apparent melting temperature ([38]; Table 1), whereas no products were observed in the reaction at 80 °C.

**Table 1:** Reported T_m(app)_ values for AA9 and AA10 LPMOs in the literature

Enzyme (and additives)^a^	*T*_m(app)_ (°C)	pH (buffer)	Method	Domains	Reversible/irreversible folding	References
mgLPMO10-*holo*	83.0	6.0 (phosphate)	DSC	AA10-CBM2	Irreversible	This study
mgLPMO10-*apo*	73.0	6.0 (phosphate)	DSC	AA10-CBM2	Irreversible	This study
mgLPMO10^CD^-*holo*	76.0	6.0 (phosphate)	DSC	AA10	Irreversible	This study
*Sc*LPMO10C-*holo*	64.1	6.0 (phosphate)	DSF	AA10-CBM2	ND	Jensen et al. [38]
*Sm*LPMO10A-*holo*	71.2	6.0 (phosphate)	DSF	AA10	ND	Jensen et al. [38]
*Sm*LPMO10A-*holo*	74.4	5.0 (acetate)	DSC	AA10	Reversible	Sugimoto et al. [45]
*Sm*LPMO10A-*apo*	65.6	5.0 (acetate)	DSC	AA10	Reversible	Sugimoto et al. [45]
*Sm*LPMO10A	70.3	8.0 (Tris–HCl)	ITF	AA10	ND	Vaaje-Kolstad et al. [46]
*Ef*LPMO10A	72.0	8.0 (Tris–HCl)	ITF	AA10	ND	Vaaje-Kolstad et al. [46]
*Ba*LPMO10A-*holo*	65.0	5.0 (acetate)	DSF	AA10	ND	Hemsworth et al. [47]
*Ba*LPMO10A-*apo*	46.0	5.0 (acetate)	DSF	AA10	ND	Hemsworth et al. [47]
*Ba*LPMO10A + EDTA	48.0	5.0 (acetate)	DSF	AA10	ND	Hemsworth et al. [47]
*Ba*LPMO10A-Zn^2+^	52.0	5.0 (acetate)	DSF	AA10	ND	Hemsworth et al. [47]
*Ba*LPMO10A-Ni^2+^	53.0	5.0 (acetate)	DSF	AA10	ND	Hemsworth et al. [47]
*Ba*LPMO10A-Mn^2+^	46.0	5.0 (acetate)	DSF	AA10	ND	Hemsworth et al. [47]
*Ta*LPMO9A-*holo*	74.3	7.0 (MOPS)	ITF	AA9	Reversible	Singh et al. [48]
*Ta*LPMO9A-*apo*	65.2	7.0 (MOPS)	ITF	AA9	Reversible	Singh et al. [48]
*Ta*LPMO9A-*holo* (deglycosylated)	67.9	7.0 (phosphate)	ITF	AA9	Reversible	Singh et al. [48]
*Mt*LPMO9B	75.0^b^	7.0 (phosphate)	CD	AA9-CBM1	Irreversible	Frommhagen et al. [49]
*Mt*LPMO9D	68.0^b^	7.0 (phosphate)	CD	AA9	Irreversible	Frommhagen et al. [49]
*Nc*LPMO9C-*holo*	61.5	6.0 (phosphate)	DSF	AA9-CBM1	ND	Kracher et al. [44]^c^
*Nc*LPMO9C-*holo*	44.0	4.0 (acetate)	DSF	AA9-CBM1	ND	Kracher et al. [44]
*Nc*LPMO9C-*holo*	34.7	4.0 (citrate)	DSF	AA9-CBM1	ND	Kracher et al. [44]
*Nc*LPMO9C-*apo* (+ EDTA)	52.7	6.0 (phosphate)	DSF	AA9-CBM1	ND	Kracher et al. [44]
*Nc*LPMO9C-*holo* + AscA	48.8	6.0 (phosphate)	DSF	AA9-CBM1	ND	Kracher et al. [44]
*Nc*LPMO9C-*holo* + xyloglucan	61.4	6.0 (phosphate)	DSF	AA9-CBM1	ND	Kracher et al. [44]
*Nc*LPMO9C-*holo* + xyloglucan + AscA	60.4	6.0 (phosphate)	DSF	AA9-CBM1	ND	Kracher et al. [44]
*Nc*LPMO9C	63.0	6.0 (phosphate)	DSC	AA9-CBM1	ND	Kittl et al. [30]
*Nc*LPMO9J	66.9	6.0 (phosphate)	DSC	AA9-CBM1	ND	Kittl et al. [30]
*Nc*LPMO9F	68.9	6.0 (phosphate)	DSC	AA9	ND	Kittl et al. [30]
*Nc*LPMO9E	67.9	6.0 (phosphate)	DSC	AA9-CBM1	ND	Kittl et al. [30]
*Pv*LPMO9A-*holo*	59.6	7.0 (Polybuffer 96, GE Healthcare)	DSC	AA9-C-term^d^	ND	Semenova et al. [50]
*Pv*LPMO9A-*apo*	49.9	7.0 (Polybuffer 96, GE Healthcare)	DSC	AA9-C-term^d^	ND	Semenova et al. [50]

Ligands or other molecules present during the determination of the apparent melting temperature are provided with the enzyme names

*ND* not detected, *DSC* differential scanning calorimetry, *DSF* differential scanning fluorimetry, *ITF* intrinsic Trp fluorescence, *CD* circular dichroism

^a^The term “holo” refers to the enzyme with copper bound, while “apo” refers to the metal free enzyme; the presence of other metals and/or substrates is indicated. If nothing is written by the enzyme name, the experiment was performed with unknown copper content

^b^Temperatures estimated based on CD diagrams in Frommhagen et al. [49]

^c^The table only shows a selection of stability data presented in the study by Kracher et al. [44]

^d^C-terminal peptide of unknown function

**Fig. 5:**
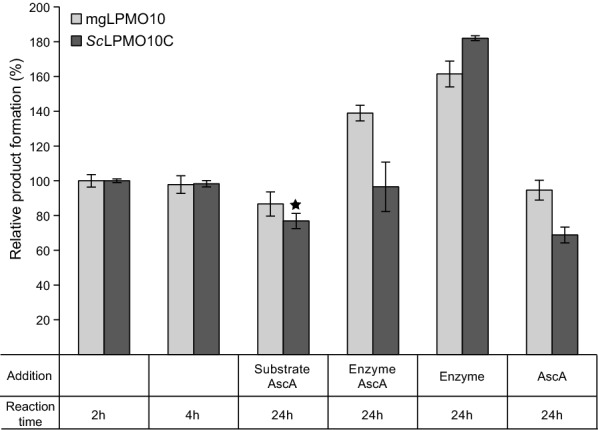
Probing early inactivation of mgLPMO10 and *Sc*LPMO10C. A “mother reaction” containing 10 g/L Avicel in 50 mM sodium phosphate pH 6.0, 1 µM mgLPMO10 (light grey) or *Sc*LPMO10C (dark grey) and 1 mM AscA was incubated at 50 °C until product formation was stable (the left two sets of bars show product formation in the “mother reaction” after 2 and 4 h). The “mother reaction” was subsequently split into four new reactions to which fresh reactants were added, as indicated in the figure, to the same final concentration as in the starting “mother reaction”. The reactions were then incubated for another 20 h before separating the soluble fraction from the insoluble particles. The solubilized products were degraded to oxidized dimers and trimers by incubation with 1 µM mgCel6A prior to product quantification. All reactions were carried out in triplicates, and error bars show ± SD (*n* = 3), except for the reaction marked with a star where *n* = 2

The product formation curves for mgLPMO10 in Fig. 4 show that the reactions slow down after 60–120 min of incubation, also when using incubation temperatures that are well below *T*_m(app)_. For *Sc*LPMO10C the product formation levels out already after 20–30 min. To evaluate the causes of the reduction in product formation, an experiment was carried out where extra reactants (i.e., substrate, enzyme and/or reductant) were added after reaching the endpoint of the reaction (Fig. 5). The results showed that the enzymes became inactivated, since the addition of more substrate and/or ascorbic acid alone did not lead to the release of more products, whereas the addition of fresh enzyme did. Thus, even at temperatures that are low (e.g., 50 °C) relative to the *T*_m(app)_ (83 °C), enzyme inactivation limits the reaction, as is commonly observed for LPMOs [22]. It is worth noting that the addition of extra ascorbic acid next to fresh enzyme was not favorable, which could be due to ascorbic acid promoting rapid inactivation of the added enzyme. In the case of mgLPMO10, an identical experiment was done at 60 °C, with similar results.

### The effect of mgLPMO10 on the efficiency of Celluclast® and individual cellulases in cellulose degradation

**Fig. 6:**
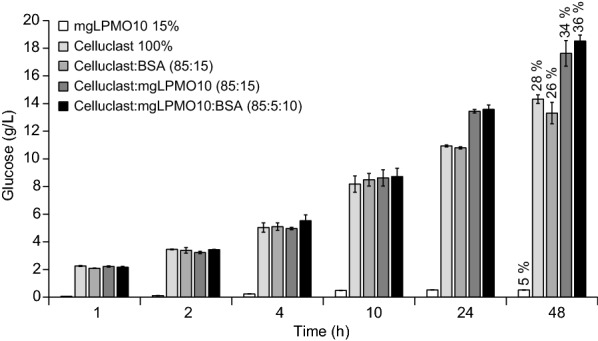
Effect of mgLPMO10 on saccharification of Avicel by Celluclast®. Reaction mixtures were incubated at 50 °C and contained 50 g/Avicel in 50 mM sodium phosphate buffer pH 6.0. The total protein dosage was 4 mg/g glucan, with 100% of Celluclast® (i.e., a mixture of Celluclast® and β-glucosidase in a ratio of 10:1), or Celluclast® and mgLPMO10 in different ratios. Samples from the reaction with mgLPMO10 alone were subjected to postdigestion with a mixture of Celluclast® and β-glucosidase prior to product analysis. BSA was used as control. The numbers over the 48 h bars show the total glucan conversion (% of maximum). All reactions contained 1 mM AscA and were run in triplicates with standard deviations indicated in the figure

Full-length mgLPMO10 was tested in a spiking experiment with the commercial cellulase cocktail Celluclast®, which has little LPMO activity [39]. In the experiment, 5 or 15% of the standard enzyme cocktail, consisting of Celluclast® and a β-glucosidase in a 10:1 ratio, was replaced by mgLPMO10, or by BSA as a control. Figure 6 shows that replacing parts of the Celluclast® cocktail with mgLPMO10 led to clearly higher saccharification yields. The positive effect of the LPMO became only visible in the later phase of the reaction, suggesting that LPMO activity is particularly important for saccharification of the more recalcitrant fraction of the cellulose substrate. Interestingly, the synergistic effect was the same for the experiments with 5% and 15% mgLPMO10, indicating that only a small fraction of LPMO is needed to boost the efficiency of the cellulase cocktail. When 15% BSA was added instead of the LPMO, a negative effect was observed at 48 h, but the effect was negligible in the beginning of the reaction.

**Fig. 7:**
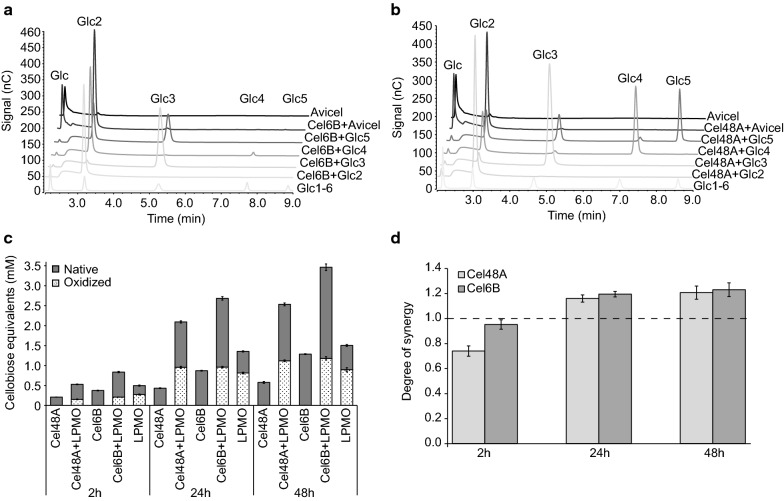
Synergy between mgLPMO10 and mgCBHs and product profiles for the mgCBHs. **a**, **b** show product profiles after degradation of cello-oligosaccharides and Avicel for mgCel6B and mgCel48A, respectively. The enzymes (0.5 µM) were incubated with 0.1 g/L cello-oligosaccharide (DP 2–5) in 50 mM sodium phosphate pH 6.0 at 60 °C. The chromatograms for the oligomeric substrates are from HPEAC-PAD analysis of samples taken after 10 min reaction time for mgCel6B and 30 min reaction time for mgCel48A. The chromatograms for Avicel degradation are the 2 h samples in the experiment shown in **c**. Note the background signal for glucose in the Avicel-only control and the fact that glucose production by the enzymes was very low. Panel **c** shows product formation over time during degradation of Avicel by various enzyme combinations. Reaction mixtures were incubated at 60 °C and contained 10 g/L Avicel in 50 mM sodium phosphate buffer pH 6.0, 1 mM AscA and 1 µM of each enzyme. Prior to quantification of native and oxidized solubilized products by HPEAC-PAD, these products were treated with mgCel6A to simplify the product mixture, and the amounts of the various products were converted to cellobiose equivalents. Error bars represent standard deviations with *n* = 3. Panel **d** shows the degree of synergy calculated from data in **c**. Error bars represent propagated standard deviations from **c**

To further explore the impact of mgLPMO10 on cellulase activity two putative CBHs derived from the same metagenome were expressed, namely mgCel6B and mgCel48A, where the latter is known to be co-expressed with mgLPMO10 [35]. MgCel48A has an N-terminal CBM2 domain followed by a domain of unknown function with immunoglobulin-like fold and a catalytic GH48 domain. The domain structure of mgCel48A is similar to that of the well-studied reducing-end directed CBH *Tf*Cel48A from *Thermobifida fusca* [40], with 58% overall sequence identity. MgCel6B has an N-terminal CBM2 followed by the GH6 catalytic domain. Its domain structure and sequence (64% identity) are similar to that of *Tf*Cel6B, which is thought to act from the non-reducing end [41, 42]. Product profiles for the two cellulases (Figure 7a, b) show that the by far dominant product generated from Avicel is cellobiose. This dominance, and the concurrent low production of glucose and cellotriose (which neither of the enzymes can cleave; Fig. 7a, b) is indicative of processive action [43]. Studies of product formation upon combining the LPMO and each of the cellulases (Fig. 7c, d) showed no synergistic effect, and even a minor antagonistic effect, in the initial phase of the reaction, whereas over time synergistic effects were detected for both CBH-LPMO combinations.

## Discussion

In this study, we describe a thermostable cellulose C1-oxidizing LPMO with an apparent melting temperature of 83 °C, which, to our knowledge, is the highest T_m(app)_ reported for an LPMO (Table 1). Indeed, this novel LPMO was capable of cellulose conversion at temperatures up to 80 °C, in contrast to mesostable LPMO10C from *S. coelicolor* (*Sc*LPMO10C), which showed impaired activity already at 70 °C. Considering *Sc*LPMO10C’s apparent T_m_ of 64 °C [38], it may seem surprising that the enzyme works at all at 70 °C, but this may be explained by stabilizing effects of substrate binding, as has been observed for fungal *Nc*LPMO9C in the presence of a xyloglucan substrate [44]. The effect of the CBM on the apparent melting temperature of mgLPMO10 is not easily explained. The linker between the CBM2 and the LPMO domain in *Sc*LPMO10C is flexible and NMR studies have shown that the two domains move independently [19]. Thus, one might expect the catalytic domain and the CBM to act as separated units that fold and unfold independently. This could result in an unfolding curve with two transitions or with a more gradual “mixed” transition, as observed previously for a CBM-containing LPMO [49]. The unfolding curve of mgLPMO10 in Fig. 3 shows only a single transition, which indicates that the two domains do not unfold independently or that they unfold independently and have approximately the same melting temperature. Based on the present data it cannot be excluded that the two domains interact with each other in a manner that leads to stabilization. However, the decrease in stability upon removal of the linker and the CBM may also be due to the actual truncation, which may have created an unfavorable configuration at the C-terminus of the mgLPMO10^CD^ variant. Nevertheless, the *T*_m(app)_ of mgLPMO10^CD^ is higher than most other reported T_m_ values for LPMOs (Table 1).

LPMO activity and stability under catalytic conditions depend on multiple factors that cannot easily be resolved. For example, we do not know the effects of elevated temperature on the reducing power and stability (reactivity with O_2_) of the reductant, nor on the various off-pathway reactions that the LPMO may engage in, in particular H_2_O_2_-generating oxidase activity (also called uncoupled reaction) and autocatalytic enzyme inactivation. Product yields and apparent enzyme inactivation do not necessarily reflect only thermal stability of the LPMOs or the general effect of temperature on enzyme catalysis. For example, increased LPMO activity at higher temperatures could reflect that H_2_O_2_ is generated at a faster rate as the temperature increases and may not necessarily reflect the catalytic rate of the LPMOs. Likewise, temperature effects on LPMO reduction and substrate binding, where the latter would affect both the oxidase activity of the LPMO and the sensitivity to autocatalytic inactivation, may partly underlie the temperature effects shown in Fig. 4. Of note, the concentrations of dissolved oxygen become less as temperature increases, but this does not seem to negatively affect initial LPMO activity. Despite these uncertainties, the present data leave no doubt that mgLPMO10 is a very stable LPMO that is active and stable at higher temperatures compared to its mesophilic counterparts, such as *Sc*LPMO10C.

The observation that adding fresh enzymes to the reactions (Fig. 5) restored activity shows that enzyme inactivation limits the reactions. The observation that simultaneous addition of more AscA is unfavorable, especially for *Sc*LPMO10C, was unexpected. This is not easily explained, but there are several side reactions that can occur and damage the enzyme [22]. In particular, addition of fresh AscA will lead to increased generation of H_2_O_2_. Increased formation of H_2_O_2_ would normally speed up the reaction, since H_2_O_2_ is a good co-substrate for LPMOs [20, 25, 28]. However, in this case, one could envisage a situation where too much H_2_O_2_ is produced due to excess AscA, which can damage the LPMO.

**Fig. 8:**
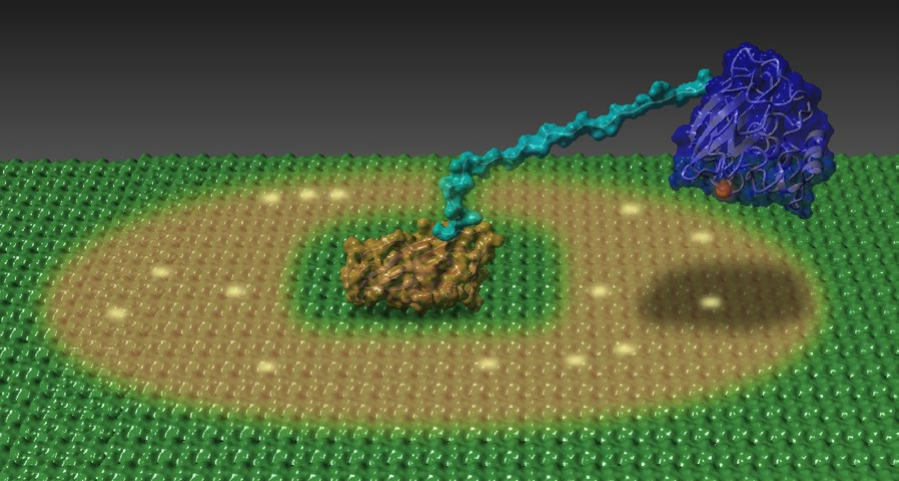
Putative model for cellulose oxidation by *Sc*LPMO10C based on NMR data. The CBM2 (orange) is docked on a cellulose surface (green) and tethered by a 30-residue linker (cyan) to the LPMO domain (blue). The brown color on the cellulose surface indicates an area of approximately 1300 Å^2^, corresponding to approximately 300 glucose residues, that the LPMO domain can reach (rough estimate based on the radius of gyration of the ensemble of NMR structures). The yellow dots indicate potential sites of oxidation (random sites, for illustration purposes). This figure and its underlying data are from Courtade et al. [19]

The observed enzyme stabilities for mgLPMO10 under catalytic conditions may reflect true folding stability (which is related to the *T*_m(app)_) and/or resistance against oxidative enzyme self-inactivation. It is well known that LPMOs may suffer from autocatalytic inactivation during catalysis [20–22] and this is also observed in the present study. The experiments depicted in Fig. 4 clearly show that maximum product levels (appr. 500 μM) obtained by mgLPMO10 at incubation temperatures that should not lead to enzyme unfolding are nevertheless limited by enzyme stability. Interestingly, the experiments of Fig. 5 also show that product yields were not limited by ascorbic acid. At a first glance this may seem strange since the product yields in this case (Fig. 4) indicate that as much as 50% of the 1 mM added ascorbic acid has been converted to soluble oxidized products and since one would expect a considerable amount of additional oxidized products that are not soluble (e.g., oxidized sites on the polymeric substrate). However, it has been shown for *Sc*LPMO10C, with a domain structure very similar to mgLPMO10 (Fig. 1) that in reactions with 10 g/L Avicel, as used here, > 90% of the generated oxidized products are soluble [19]. Thus, it seems possible to generate 500 μM soluble oxidized products without consuming the 1 mM of reductant. The high fraction of soluble products is due to the fact that the CBM “immobilizes” the LPMO on the substrate surface, thus increasing the chance of the same polymer being cleaved twice, which is a prerequisite for generating a high fraction of soluble products (see Fig. 8 and Courtade et al. [19] for further discussion).

It is worth noting the difference in the approximate maximum product levels obtained in reactions with *Sc*LPMO10C (200 μM) and mgLPMO10 (500 μM). This could indicate that the more thermally stable mgLPMO10 is also more resistant against oxidative damage (as also suggested by the effects of adding AscA in the experiments depicted in Fig. 5; see above). In this respect, it may be relevant that the linker connecting the catalytic domain and the CBM2 domain is seven residues longer in mgLPMO10. Studies on *Sc*LPMO10C have shown that the catalytic domain and the CBM move independently relative to each other, and that, due to the anchoring of the CBM2 on the cellulose surface, the freely moving but still restricted catalytic domain makes multiple cuts in nearby cellulose chains [19]. In the previous study of *Sc*LPMO10C it was estimated that a cellulose-anchored catalytic domain could reach some 1300 Å^2^ of cellulose surface (Fig. 8). An extension of the linker by seven residues, perhaps adopting an extended conformation, could considerably increase this sampling area, which would mean that mgLPMO10 can make more cuts per binding event and will, thus, spend less time in solution per number of cuts. Since reduced non-substrate-bound LPMOs are prone to inactivation through reactions with O_2_ or H_2_O_2_, spending less time in solution per number of cuts, or, phrased differently, staying in close proximity of the substrate for a longer time, will reduce oxidative damage and improve enzyme stability.

Combining mgLPMO10 with Celluclast®/β-glucosidase (Fig. 6) resulted in clearly improved saccharification yields in reactions with Avicel. The studies with individual enzymes (Fig. 7) showed that both mgCel48A, presumably acting from the reducing-end, and mgCel6B, presumably acting from the non-reducing-end, also act in synergy with mgLPMO10, however the synergy is only observed over time and the synergistic effect is minor. It is worth noting that oxidized products are a major part of the total product formation in these reactions. Limited synergistic effects between LPMOs and individual cellulases have been observed before. For example, earlier work [51, 52] has shown limited synergistic and even inhibitory effects upon combining a C1-oxidizing LPMO with the well-studied reducing-end active CBH from *Trichoderma reesei* (*Tr*Cel7A). Further studies are needed to disclose the exact nature of the cellulase—LPMO interplay and to analyze to what extend and how the regioselectivity of the LPMO may affect the type of cellulase it synergizes with. Such studies should include multiple substrates and varying cellulase-LPMO ratios, since it is well known that the degree of cellulase-LPMO synergy is dependent in these factors [51–53].

The synergistic effects observed when adding mgLPMO10 to Celluclast® are encouraging. In a study by Müller et al. [39] it was reported that addition of various amounts of the well-studied C1/C4 oxidizing fungal LPMO *Ta*LPMO9A boosted the degradation of cellulose in steam exploded birch by Celluclast®/β-glucosidase. In both this previous and the present study, the maximum improvement in saccharification yield amounted to 30–40%, but to achieve this 15% LPMO needed to be added in the birch study, as opposed to only 5% (or less) in the present study with Avicel. While the two studies are not directly comparable, for example because of the different contents of reducing power (lignin in the birch experiment, 1 mM ascorbic acid in the present study), it seems safe to conclude that mgLPMO10 is a powerful enzyme.

## Conclusion

This study describes a thermostable LPMO, which, to our knowledge, possesses the highest melting temperature reported for an LPMO and which, uniquely, can carry out oxidative cleavage of cellulose at 80 °C. The use of thermostable enzymes in biorefineries is of interest, since it is often preferable to run processes at high temperatures. MgLPMO10, with its profound boosting effect on cellulase efficiency that is comparable to the effect of commonly used fungal LPMOs, is a good candidate to be considered for the development of thermostable enzyme cocktails for industrial processing of lignocellulosic biomass.

## Methods

### LPMO expression and purification

The mgLPMO10-encoding gene originates from publicly available metagenome data accessible in the Joint Genome Institute IMG/M database (https://img.jgi.doe.gov/cgi-bin/m/main.cgi; IMG genome ID 2199352008). The microbial community was sampled from rice straw that had been inoculated with compost and incubated at 55 °C. Metagenomic and metatranscriptomic analyses of this community are available [35, 54]. The 1092 bp gene encoding mgLPMO10 (IMG/M gene ID 2200500718) was detected as overexpressed in the community ([35]; note that the LPMO is referred to as a CBM33 in this paper).

The *mglpmo10* encoding gene (codon-optimized for *Escherichia coli* expression) was synthesized (GenScript, Piscataway, NJ, USA) and nucleotides 94–1089 (omitting the predicted 31 amino acid signal peptide sequence and the stop codon) were cloned in the pRSET B expression vector (Invitrogen, Carlsbad, California, USA), already containing the well-functioning signal sequence of *Sm*LPMO10A (also known as CBP21), following the protocol described by Forsberg et al. [36]. The forward primer employed to amplify and insert the gene into the pRESET B vector was 5′-CGCAACAGGCGAATGCCCACGGTGCGGCGATGGT-3′ and the reverse primer was 5′-CAGCCGGATCAAGCTT**TTA**CGCGCTGGTGCAGGTC-3′. For production of the catalytic domain alone (mgLPMO10^CD^; nucleotide 94–669) the reverse primer 5′-CAGCCGGATCAAGCTT**TTA**ATCAAAAACCACGTCGCT-3′ was used instead. The underlined nucleotides represent an over-hang sequence with the pRSET B vector for fusing the gene in the pRSET B vector using the In-Fusion HD cloning kit (Clontech, Mountain View, California, USA), and the “TTA” sequence of the reverse primers (in bold) encodes a stop codon at the end of the LPMO-encoding sequence. The gene sequences were verified by Sanger sequencing (Eurofins GATC, Cologne, Germany).

Chemically competent OneShot BL21 Star™ (DE3) *E. coli* cells (Invitrogen) harboring a pRSET B vector with an LPMO-encoding gene were used to inoculate 500 mL Terrific Broth (TB) supplemented with 100 µg/mL ampicillin, followed by incubation at 37 °C for 20 h in a Harbinger system (Harbinger Biotechnology & Engineering, Markham, Canada). Note that expression was driven by leakiness of the T7 promoter and that no inducer molecule was added. Cells were harvested by centrifugation at 5000×*g* for 15 min at 4 °C (Beckman Coulter Brea, California, USA), after which a periplasmic extract was prepared using an osmotic shock method [55].

Periplasmic extracts were filtered using 0.45 µm syringe filters (Sarstedt, Nümbrecht, Germany) and adjusted to 50 mM Tris–HCl (pH 8.0) after which the proteins were purified by anion-exchange chromatography using an ÄKTA pure chromatography system (GE Healthcare, Chicago, USA) equipped with a 5-ml HiTrap DEAE FF column (GE Healthcare). As running buffer, 50 mM Tris–HCl (pH 8.0) was used and proteins were eluted using a linear salt gradient (0–500 mM NaCl) over 60 column volumes. MgLPMO10 and mgLPMO10^CD^ displayed poor binding to the DEAE column, and thus mainly ended up in the flow through. Protein fractions were examined by SDS-PAGE (Bio-Rad, Hercules, California, USA) and relevant fractions (i.e., the flow through in this case) were concentrated to 1 mL using 10,000 MWCO (molecular weight cut-off) Vivaspin ultrafiltration tubes (Sartorius, Göttingen, Germany).

The concentrated protein samples were used in a second purification step by size exclusion chromatography, using a HiLoad 16/60 Superdex 75 column with a running buffer consisting of 50 mM Tris–HCl (pH 8.0) and 200 mM NaCl. Fractions containing the LPMO, as determined by SDS-PAGE, were concentrated using 10,000 MWCO Vivaspin ultrafiltration tubes (Sartorius) with simultaneous buffer exchange to 20 mM sodium phosphate buffer (pH 6.0).

Prior to use, purified enzymes were incubated for 30 min at room temperature with Cu(II)SO_4_ in a 1:3 molar ratio (LPMO:Cu^2+^) to generate copper-saturated LPMO. Apo enzyme was prepared by incubating the purified enzyme for 30 min at room temperature with ethylenediaminetetraacetic acid (EDTA) in a 1:10 molar ratio (LPMO:EDTA). A PD Midi-Trap G-25 column (GE Healthcare) was subsequently used to desalt and remove excess copper or EDTA from the enzyme.

Protein concentrations were determined by measuring A_280_ (absorbance at 280 nm) in a spectrophotometer (Eppendorf Biophotometer, Eppendorf, Hamburg, Germany) and absorbances were converted to protein concentrations using theoretical extinction coefficients calculated with the ExPASy ProtParam tool (https://web.expasy.org/protparam/). The proteins were stored at 4 °C until further use.

### Production of additional enzymes

Full-length *Sc*LPMO10C and mgCel6A endoglucanase were produced and purified as described previously by Forsberg et al. [36] and Jensen et al. [56], respectively.

Genes encoding two putative cellobiohydrolases (CBHs), namely a 101.6 kDa GH48 (mgCel48A; IMG/M gene ID 2200387045; overexpressed together with mgLPMO10 in the thermophilic compost/rice straw community [35]) and a 61.4 kDa GH6 (mgCel6B; IMG/M gene ID 2200705178) from the same metagenome [54], were synthesized (GenScript, Piscataway, NJ, USA) and cloned for intracellular expression, without the native signal peptide and with a C-terminal His-tag according to the procedure described for the endoglucanase mgCel6A from the same metagenome [56]. The genes were cloned in the pNIC-CH expression vector using 5′-TTAAGAAGGAGATATACTATGGCACCGGCATGTGAAGTTACCTAT-3′ as the forward primer and 5′-AATGGTGGTGATGATGGTGCGCACCAATCAGACGATCATAATCGGC-3′ as reverse primer for mgCel48A. The forward primer for mgCel6B, was 5′-TTAAGAAGGAGATATACTATGGCATTTGCAGCACCGGGTTGTAGC-3′, while the reverse primer was 5′-AATGGTGGTGATGATGGTGCGCCAGAGGCGGATATGCATTATCCAT-3′. The underlined nucleotides represent an over-hang sequence for ligase-independent cloning in the pNIC expression vector. The proteins were produced and purified as described for mgCel6A by Jensen et al. [56].

### Commercial cellulase cocktail

Celluclast® 1.5 L, a commercial cellulase cocktail with little LPMO activity [39] was provided by Novozymes (Bagsværd, Denmark). The Celluclast® cocktail was first mixed with a β-glucosidase, kindly supplied by Novozymes, in a 10:1 ratio (on a protein basis) to facilitate degradation of solubilized oligomers to the monomer (glucose).

### Substrates

Evaluation of LPMO oxidative activity and saccharification experiments were carried out with the microcrystalline cellulosic substrate Avicel PH-101 (Sigma-Aldrich, Darmstadt, Germany) and phosphoric acid-swollen cellulose (PASC). PASC was prepared from Avicel PH-101 as described by [57]. Cello-oligosaccharides DP3-5 were purchased from Megazyme (Bray, Ireland). Glucose and cellobiose were purchased from Sigma-Aldrich.

### Apparent melting temperature

A Nano-Differential Scanning Calorimeter III (Calorimetry Sciences Corporation, Lindon, USA) was used to determine *T*_m(app)_ of the mgLPMO10 variants. Approximately 0.4 mg/mL LPMO in 50 mM sodium phosphate buffer pH 6.0 (filtered and degassed) was heated from 25 °C to 100 °C at 1 °C/min. Buffer baselines were recorded and subtracted from the protein scans. The data were analyzed with NanoAnalyze software (https://www.tainstruments.com).

### Activity assays

Unless stated otherwise, reactions were performed in 50 mM sodium phosphate buffer (pH 6.0) at 800 rpm in an Eppendorf Thermomixer. All reactions were performed in triplicates using 2 mL micro tubes with screw cap and O-rings to avoid evaporation.

For the initial verification of oxidative cellulolytic activity, 1 µM mgLPMO10 was incubated with 5 g/L PASC or 10 g/L Avicel in the presence of 1 mM ascorbic acid (reductant) for 24 h, at 60 °C. The supernatant from PASC samples were analyzed by MALDI-ToF MS and the supernatant from Avicel samples were analyzed by HPAEC-PAD (see below) for detection of oxidized cello-oligomers and determination of LPMO regioselectivity.

Degradation of Avicel (10 g/L) was analyzed at different temperatures ranging from 40–80 °C using reaction mixtures containing 1 μM LPMO and 1 mM ascorbic acid. Aliquots were withdrawn at selected time points and filtered (0.45 μm) before being merged with an identical volume of a solution containing 2 µM endoglucanase (mgCel6A; [56]) followed by static incubation overnight at 37 °C. As a result of this procedure, all solubilized oxidized products were converted to oxidized cellobiose and oxidized cellotriose, which simplified product quantification. The products were analyzed using HPAEC-PAD (see below).

Inactivation of mgLPMO10 and *Sc*LPMO10C during cellulose degradation reactions was investigated by incubating 1 µM enzyme with 10 g/L Avicel and 1 mM AscA at 50 or 60 °C until product formation stopped. The reactions were subsequently split into four new reactions and more AscA and substrate, AscA and enzyme, only enzyme, or only AscA was added to the same final concentration as in the original reaction. The reactions were further incubated for 20 h before separating the soluble fraction from the insoluble particles by filtration (0.45 µm). The solubilized oxidized products were degraded to oxidized dimers and trimers by incubation with 1 µM mgCel6A prior to product quantification, as described above, before product analysis by HPAEC-PAD (see below).

Degradation of individual cello-oligosaccharides by mgCel48A and mgCel6B was analyzed by incubating 0.1 g/L cello-oligosaccharide (Glc_2-5_) with 0.1 µM enzyme in 50 mM sodium phosphate pH 6.0. Reactions involving mgCel48A were supplemented with 1 mM CaCl_2_ as this is known to stabilize other GH48s at high temperatures [58, 59]. Samples taken after 30 min for mgCel48A and 10 min for mgCel6B were mixed with an equal volume of 0.2 M NaOH to stop the enzyme activity. Products were analyzed by HPEAC-PAD (see below).

### Synergy experiments

Investigation of the synergistic relationship between mgLPMO10 and two putative cellobiohydrolases (CBHs; mgCel48A and mgCel6B) was performed using 10 g/L Avicel as substrate, which was subjected to degradation by 1 µM mgLPMO10 and/or 1 µM of mgCel48A or mgCel6B. The reactions contained 50 mM sodium phosphate buffer (pH 6.0), and 1 mM ascorbic acid. Reactions involving mgCel48A were supplemented with 1 mM CaCl_2_. Samples were withdrawn after 2, 24, and 48 h of incubation at 60 °C. After filtration (0.45 µm) to remove insoluble substrate, the samples were mixed with and equal volume of 2 µM mgCel6A and incubated statically at 37 °C overnight to simplify product quantification as described above. Native and oxidized cello-oligosaccharide products were analyzed using HPAEC-PAD (see below). Quantified products (cellobiose, GlcGlc1A, Glc_2_Glc1A, as well as minor amounts of glucose and cellotriose) were converted to cellobiose equivalents.

The effect of mgLPMO10 on the saccharification efficiency of a mixture of Celluclast® and a β-glucosidase (see above) was investigated using 50 g/L Avicel as substrate. The total protein load was 4 mg per gram glucan (the glucan content of Avicel is 92.2%; [39]). The reactions contained 50 mM sodium phosphate, pH 6.0, and 1 mM ascorbic acid, and were incubated at 50 °C (optimal temperature for Celluclast®). Samples were withdrawn at selected timepoints and the supernatants were separated from the insoluble substrate by filtration over a 0.45 μm microtiter filter plates and kept at -20 °C until product analysis. Samples from reactions with mgLPMO10 alone were treated with an equal volume of a 0.36 g/L mixture of Celluclast® and a β-glucosidase to convert oxidized cello-oligosaccharides to glucose and gluconic acid. Products were analyzed using UHPLC (see below).

### Product analysis with HPAEC-PAD

Oxidized products were detected by high-performance anion-exchange chromatography (HPAEC) using a Dionex™ ICS5000 or ICS3000 (only for data in Fig. 2b) system (Thermo Scientific, Sunnyvale, CA) equipped with a disposable electrochemical gold electrode and a CarboPac PA1 (2 × 250 mm) or a CarboPac PA200 (3 × 250 mm) column (Dionex, Sunnyvale, CA, USA) operated with 0.1 M NaOH (eluent A) at a column temperature of 30 °C [60] and a flow rate of 0.25 ml/min or 0.5 ml/min for the PA1 column and PA200 column, respectively. A multistep linear gradient with increasing amounts of eluent B (0.1 M NaOH + 1 M NaOAc) was used to elute the products. For the PA1 column the gradient was: 0–10% B over 10 min; 10–18% B over 10 min; 18–30% B over 1 min; 30–100% B over 1 min; 100–0% B over 0.1 min; 0% B (reconditioning) for 13.9 min. For the PA200 column the gradient was: 0–10% B over 5 min; 10–18% B over 5 min; 18–30% B over 0.5 min; 30–100% B over 0.5 min; 100–0% B over 0.05 min; 0% B (reconditioning) for 6.95 min. The gradient used for the ICS3000 system (PA1 column) was: 0–10% B over 10 min; 10–30% B over 25 min; 30–100% B over 5 min; 100–0% B over 1 min; 0% B (reconditioning) for 9 min. Data collection and analysis were carried out with the Chromeleon 7.0 software. Cellobiose (Sigma Aldrich) and cellotriose (Megazyme, Bray, Ireland) were used as substrates for production of C1-oxidized standards, i.e., cellobionic acid (GlcGlc1A) and cellotrionic acid (Glc_2_Glc1A), respectively, by treatment with cellobiose dehydrogenase from *Myriococcum thermophilum* (*Mt*CDH) [61].

Native (Glc_1-6_) and oxidized (GlcGlc1A and Glc_2_Glc1A) cello-oligosaccharides resulting from reactions with CBHs were analyzed using the same HPAEC system and the same eluents as described above, with a CarboPac PA200 column. The gradient was: 0–5.5% B over 3 min; 5.5–15% B over 6 min; 15–100% B over 11 min; 100–0% B over 0.1 min; 0% B (reconditioning) for 5.9 min.

### Product analysis with UHPLC

Quantification of glucose resulting from reactions with the Celluclast® enzyme cocktail was achieved using a Dionex Ultimate 3000 UHPLC system (Dionex, Sunnyvale, CA, USA) equipped with a Rezex ROA-Organic Acid H + (8%), 300 × 7.8 mm analytical column and a SecureGuard Carbo-H + 4 × 3.0 mm guard column (Phenomenex, Torrance, CA, USA) operated at 65 °C. Sample components were eluted isocratically over 22 min using 5 mM sulfuric acid as mobile phase with a flow rate of 0.6 mL/min. Products were detected using a refractive index (RI) detector 101 (Shodex, Tokyo, Japan) and data collection and analysis were carried out with the Chromeleon 7.0 software.

### MALDI-ToF product analysis

Products in reaction supernatants were assayed qualitatively using a matrix-assisted laser desorption/ionization time-of-flight (MALDI-ToF) UltrafleXtreme mass spectrometer (Bruker Daltonics GmbH, Bremen, Germany) equipped with a Nitrogen 337-nm laser. Reaction mixtures (1 µL) were applied to an MTP 384 ground steel target plate TF (Bruker Daltonics) and mixed with 2 µL of 9 mg/ml of 2,5-dihydroxybenzoic acid dissolved in 30% acetonitrile, followed by air-drying. Data collection and analysis were carried out using the Bruker FlexAnalysis software.

## Data Availability

All appropriate data for this study have been included in the manuscript.

## References

[1] Pauly M, Keegstra K (2008). Cell-wall carbohydrates and their modification as a resource for biofuels. Plant J.

[2] Klemm D, Heublein B, Fink H-P, Bohn A (2005). Cellulose: Fascinating biopolymer and sustainable raw material. Angew Chem Int Ed.

[3] Duchesne LC, Larson DW (1989). Cellulose and the evolution of plant life. Bioscience.

[4] Somerville C, Bauer S, Brininstool G, Facette M, Hamann T, Milne J (2004). Toward a systems approach to understanding plant cell walls. Science.

[5] Merino ST, Cherry J, Olsson L (2007). Progress and challenges in enzyme development for biomass utilization. Biofuels. Advances in biochemical engineering/biotechnology.

[6] Harris PV, Xu F, Kreel NE, Kang C, Fukuyama S (2014). New enzyme insights drive advances in commercial ethanol production. Curr Opin Chem Biol.

[7] Davies G, Henrissat B (1995). Structures and mechanisms of glycosyl hydrolases. Structure.

[8] Horn SJ, Vaaje-Kolstad G, Westereng B, Eijsink VGH (2012). Novel enzymes for the degradation of cellulose. Biotechnol Biofuels..

[9] Vaaje-Kolstad G, Westereng B, Horn SJ, Liu Z, Zhai H, Sørlie M (2010). An oxidative enzyme boosting the enzymatic conversion of recalcitrant polysaccharides. Science.

[10] Chylenski P, Bissaro B, Sørlie M, Røhr AK, Várnai A, Horn SJ (2019). Lytic polysaccharide monooxygenases in enzymatic processing of lignocellulosic biomass. ACS Catalysis.

[11] Eibinger M, Ganner T, Bubner P, Rošker S, Kracher D, Haltrich D (2014). Cellulose surface degradation by a lytic polysaccharide monooxygenase and its effect on cellulase hydrolytic efficiency. J Biol Chem.

[12] Johansen KS (2016). Lytic polysaccharide monooxygenases: The microbial power tool for lignocellulose degradation. Trends Plant Sci.

[13] Müller G, Chylenski P, Bissaro B, Eijsink VGH, Horn SJ (2018). The impact of hydrogen peroxide supply on LPMO activity and overall saccharification efficiency of a commercial cellulase cocktail. Biotechnol Biofuels.

[14] Levasseur A, Drula E, Lombard V, Coutinho PM, Henrissat B (2013). Expansion of the enzymatic repertoire of the CAZy database to integrate auxiliary redox enzymes. Biotechnol Biofuels.

[15] Tandrup T, Frandsen KEH, Johansen KS, Berrin J-G, Lo LL (2018). Recent insights into lytic polysaccharide monooxygenases (LPMOs). Biochem Soc Trans.

[16] Phillips CM, Beeson WT, Cate JH, Marletta MA (2011). Cellobiose dehydrogenase and a copper-dependent polysaccharide monooxygenase potentiate cellulose degradation by *Neurospora crassa*. ACS Chem Biol.

[17] Quinlan RJ, Sweeney MD, Leggio LL, Otten H, Poulsen J-CN, Johansen KS (2011). Insights into the oxidative degradation of cellulose by a copper metalloenzyme that exploits biomass components. Proc Natl Acad Sci..

[18] Isaksen T, Westereng B, Aachmann FL, Agger JW, Kracher D, Kittl R (2014). A C4-oxidizing lytic polysaccharide monooxygenase cleaving both cellulose and cello-oligosaccharides. J Biol Chem.

[19] Courtade G, Forsberg Z, Heggset EB, Eijsink VGH, Aachmann FL (2018). The carbohydrate-binding module and linker of a modular lytic polysaccharide monooxygenase promote localized cellulose oxidation. J Biol Chem.

[20] Bissaro B, Røhr AK, Muller G, Chylenski P, Skaugen M, Forsberg Z (2017). Oxidative cleavage of polysaccharides by monocopper enzymes depends on H_2_O_2_. Nat Chem Biol.

[21] Loose JSM, Arntzen MØ, Bissaro B, Ludwig R, Eijsink VGH, Vaaje-Kolstad G (2018). Multipoint precision binding of substrate protects lytic polysaccharide monooxygenases from self-destructive off-pathway processes. Biochemistry.

[22] Eijsink VGH, Petrovic D, Forsberg Z, Mekasha S, Røhr ÅK, Várnai A (2019). On the functional characterization of lytic polysaccharide monooxygenases (LPMOs). Biotechnol Biofuels.

[23] Beeson WT, Phillips CM, Cate JHD, Marletta MA (2012). Oxidative cleavage of cellulose by fungal copper-dependent polysaccharide monooxygenases. J Am Chem Soc.

[24] Kjaergaard CH, Qayyum MF, Wong SD, Xu F, Hemsworth GR, Walton DJ (2014). Spectroscopic and computational insight into the activation of O_2_ by the mononuclear Cu center in polysaccharide monooxygenases. Proc Natl Acad Sci.

[25] Kuusk S, Bissaro B, Kuusk P, Forsberg Z, Eijsink VGH, Sørlie M (2018). Kinetics of H_2_O_2_-driven degradation of chitin by a bacterial lytic polysaccharide monooxygenase. J Biol Chem.

[26] Hangasky JA, Iavarone AT, Marletta MA (2018). Reactivity of O_2_ versus H_2_O_2_ with polysaccharide monooxygenases. Proc Natl Acad Sci.

[27] Bissaro B, Kommedal E, Røhr ÅK, Eijsink VGH (2020). Controlled depolymerization of cellulose by light-driven lytic polysaccharide oxygenases. Nat commun.

[28] Filandr F, Man P, Halada P, Chang H, Ludwig R, Kracher D (2020). The H_2_O_2_-dependent activity of a fungal lytic polysaccharide monooxygenase investigated with a turbidimetric assay. Biotechnol Biofuels.

[29] Jones SM, Transue WJ, Meier KK, Kelemen B, Solomon EI (2020). Kinetic analysis of amino acid radicals formed in H_2_O_2_-driven CuI LPMO reoxidation implicates dominant homolytic reactivity. Proc Natl Acad Sci.

[30] Kittl R, Kracher D, Burgstaller D, Haltrich D, Ludwig R (2012). Production of four *Neurospora crassa* lytic polysaccharide monooxygenases in *Pichia pastoris* monitored by a fluorimetric assay. Biotechnol Biofuels.

[31] Forsberg Z, Sørlie M, Petrović D, Courtade G, Aachmann FL, Vaaje-Kolstad G (2019). Polysaccharide degradation by lytic polysaccharide monooxygenases. Curr Opin Struct Biol.

[32] Haki G, Rakshit S (2003). Developments in industrially important thermostable enzymes: a review. Bioresour Technol.

[33] Tanghe M, Danneels B, Last M, Beerens K, Stals I, Desmet T (2017). Disulfide bridges as essential elements for the thermostability of lytic polysaccharide monooxygenase LPMO10C from *Streptomyces coelicolor*. Protein Eng Des Sel.

[34] Lo Leggio L, Weihe CD, Poulsen J-CN, Sweeney M, Rasmussen F, Lin J (2018). Structure of a lytic polysaccharide monooxygenase from *Aspergillus fumigatus* and an engineered thermostable variant. Carbohydr Res..

[35] Simmons CW, Reddy AP, D’haeseleer P, Khudyakov J, Billis K, Pati A (2014). Metatranscriptomic analysis of lignocellulolytic microbial communities involved in high-solids decomposition of rice straw. Biotechnol Biofuels..

[36] Forsberg Z, Mackenzie AK, Sørlie M, Røhr ÅK, Helland R, Arvai AS (2014). Structural and functional characterization of a conserved pair of bacterial cellulose-oxidizing lytic polysaccharide monooxygenases. Proc Natl Acad Sci.

[37] Forsberg Z, Vaaje-Kolstad G, Westereng B, Bunæs AC, Stenstrøm Y, MacKenzie A (2011). Cleavage of cellulose by a CBM33 protein. Protein Sci.

[38] Jensen MS, Klinkenberg G, Bissaro B, Chylenski P, Vaaje-Kolstad G, Kvitvang HF (2019). Engineering chitinolytic activity into a cellulose-active lytic polysaccharide monooxygenase provides insights into substrate specificity. J Biol Chem.

[39] Müller G, Várnai A, Johansen KS, Eijsink VGH, Horn SJ (2015). Harnessing the potential of LPMO-containing cellulase cocktails poses new demands on processing conditions. Biotechnol Biofuels.

[40] Irwin DC, Zhang S, Wilson DB (2000). Cloning, expression and characterization of a family 48 exocellulase, Cel48A, from *Thermobifida fusca*. Eur J Biochem.

[41] Zhang S, Lao G, Wilson DB (1995). Characterization of a *Thermomonospora fusca* exocellulase. Biochemistry.

[42] Wu M, Bu L, Vuong TV, Wilson DB, Crowley MF, Sandgren M (2013). Loop motions important to product expulsion in the *Thermobifida fusca* glycoside hydrolase family 6 cellobiohydrolase from structural and computational studies. J Biol Chem.

[43] Horn SJ, Sørlie M, Vårum KM, Väljamäe P, Eijsink VGH (2012). Measuring processivity. Methods Enzymol.

[44] Kracher D, Andlar M, Furtmüller PG, Ludwig R (2018). Active-site copper reduction promotes substrate binding of fungal lytic polysaccharide monooxygenase and reduces stability. J Biol Chem.

[45] Sugimoto H, Nakajima Y, Motoyama A, Katagiri E, Watanabe T, Suzuki K (2020). Unfolding of CBP21, a lytic polysaccharide monooxygenase, without dissociation of its copper ion cofactor. Biopolymers.

[46] Vaaje-Kolstad G, Bohle LA, Gaseidnes S, Dalhus B, Bjoras M, Mathiesen G (2012). Characterization of the chitinolytic machinery of *Enterococcus faecalis* V583 and high-resolution structure of its oxidative CBM33 enzyme. J Mol Biol.

[47] Hemsworth GR, Taylor EJ, Kim RQ, Gregory RC, Lewis SJ, Turkenburg JP (2013). The copper active site of CBM33 polysaccharide oxygenases. J Am Chem Soc.

[48] Singh RK, Blossom BM, Russo DA, van Oort B, Croce R, Jensen PE (2019). Thermal unfolding and refolding of a lytic polysaccharide monooxygenase from *Thermoascus aurantiacus*. RSC Adv.

[49] Frommhagen M, Westphal AH, Hilgers R, Koetsier MJ, Hinz SWA, Visser J (2018). Quantification of the catalytic performance of C1-cellulose-specific lytic polysaccharide monooxygenases. Appl Microbiol Biotechnol.

[50] Semenova MV, Gusakov AV, Telitsin VD, Rozhkova AM, Kondratyeva EG, Sinitsyn AP (2020). Purification and characterization of two forms of the homologously expressed lytic polysaccharide monooxygenase (PvLPMO9A) from *Penicillium verruculosum*. Biochim Biophys Acta.

[51] Zhou H, Li T, Yu Z, Ju J, Zhang H, Tan H (2019). A lytic polysaccharide monooxygenase from *Myceliophthora thermophila* and its synergism with cellobiohydrolases in cellulose hydrolysis. Int J Biol Macromol.

[52] Tokin R, Ipsen JØ, Westh P, Johansen KS (2020). The synergy between LPMOs and cellulases in enzymatic saccharification of cellulose is both enzyme-and substrate-dependent. Biotechnol Lett.

[53] Hu J, Arantes V, Pribowo A, Gourlay K, Saddler JN (2014). Substrate factors that influence the synergistic interaction of AA9 and cellulases during the enzymatic hydrolysis of biomass. Energy Environ Sci.

[54] Reddy AP, Simmons CW, D’haeseleer P, Khudyakov J, Burd H, Hadi M (2013). Discovery of microorganisms and enzymes involved in high-solids decomposition of rice straw using metagenomic analyses. PLoS ONE.

[55] Manoil C, Beckwith J (1986). A genetic approach to analyzing membrane protein topology. Science.

[56] Jensen MS, Fredriksen L, MacKenzie AK, Pope PB, Leiros I, Chylenski P (2018). Discovery and characterization of a thermostable two-domain GH6 endoglucanase from a compost metagenome. PLoS ONE.

[57] Wood TM, Wood WA, Kellogg ST (1988). Preparation of crystalline, amorphous, and dyed cellulase substrates. Methods Enzymol. Biomass Part A: Cellulose and Hemicellulose.

[58] Bronnenmeier K, Rücknagel KP, Staudenbauer WL (1991). Purification and properties of a novel type of exo-1, 4-β-glucanase (Avicelase II) from the cellulolytic thermophile *Clostridium stercorarium*. Eur J Biochem.

[59] Morag E, Halevy I, Bayer EA, Lamed R (1991). Isolation and properties of a major cellobiohydrolase from the cellulosome of *Clostridium thermocellum*. J Bacteriol.

[60] Westereng B, Agger JW, Horn SJ, Vaaje-Kolstad G, Aachmann FL, Stenstrøm YH (2013). Efficient separation of oxidized cello-oligosaccharides generated by cellulose degrading lytic polysaccharide monooxygenases. J Chromatogr A.

[61] Zámocký M, Schümann C, Sygmund C, O’Callaghan J, Dobson AD, Ludwig R (2008). Cloning, sequence analysis and heterologous expression in *Pichia pastoris* of a gene encoding a thermostable cellobiose dehydrogenase from *Myriococcum thermophilum*. Protein Expr Purif.

